# Redetermination of di-μ-hydrido-hexa­hydridotetra­kis(tetra­hydro­furan)dialuminium(III)magnesium(II)

**DOI:** 10.1107/S1600536810014200

**Published:** 2010-04-28

**Authors:** Hima Kumar Lingam, Xuenian Chen, Teshome Yisgedu, Zhenguo Huang, Ji-Cheng Zhao, Sheldon G. Shore

**Affiliations:** a Department of Materials Science and Engineering, The Ohio State University, Columbus, OH 43210, USA; b Department of Chemistry, The Ohio State University, Columbus, OH 43210, USA

## Abstract

The structure of the title compound, [Mg(AlH_4_)_2_(C_4_H_8_O)_4_], has been redetermined at 150 K. The Mg^II^ ion is hexa­coordinated to four tetra­hydro­furan (THF) ligands, and two AlH_4_^−^ anions through bridging H atoms. The Al—H distances are more precise compared to those previously determined [Nöth *et al.* (1995 ▶). *Chem. Ber*. **128**, 999–1006; Fichtner & Fuhr (2002 ▶). *J. Alloys Compd*, **345**, 386–396]. The mol­ecule has twofold rotation symmetry.

## Related literature

For the synthesis of Mg(AlH_4_)_2_·4THF, see: Ashby *et al.* (1970 ▶); Shen & Che (1991 ▶); Nöth *et al.* (1995 ▶). For the synthesis of AlH_4_MgBH_4_, see: Ashby & Goel (1977 ▶). For previous determinations of the crystal structure of Mg(AlH_4_)_2_·4THF, see: Noth *et al.* (1995 ▶); Fichtner & Fuhr (2002 ▶). For the thermal decomposition properties of Mg(AlH_4_)_2_·4THF, see: Dilts & Ashby (1972 ▶). For other alanate structures, see: Sklar & Post (1967 ▶); Lauher *et al.* (1979 ▶); Fichtner & Fuhr (2002 ▶); Fichtner *et al.* (2004 ▶).
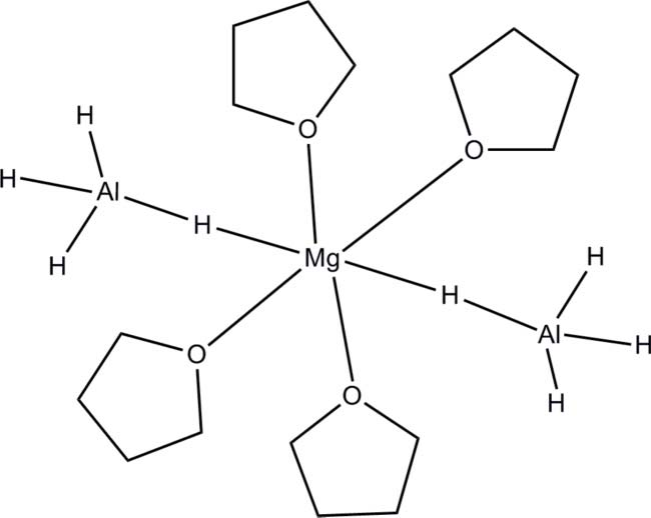


## Experimental

### 

#### Crystal data

[Al_2_MgH_8_(C_4_H_8_O)_4_]
*M* *_r_* = 374.75
Orthorhombic, 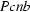

*a* = 10.161 (2) Å
*b* = 14.027 (3) Å
*c* = 16.429 (3) Å
*V* = 2341.6 (8) Å^3^
*Z* = 4
Mo *K*α radiation
μ = 0.16 mm^−1^
*T* = 150 K
0.38 × 0.31 × 0.19 mm

#### Data collection

Nonius Kappa CCD diffractometer
Absorption correction: multi-scan (*SCALEPACK*; Otwinowski & Minor, 1997 ▶) *T*_min_ = 0.940, *T*_max_ = 0.969
5018 measured reflections
2687 independent reflections
1973 reflections with *I* > 2σ(*I*)
*R*_int_ = 0.017

#### Refinement

*R*[*F* ^2^ > 2σ(*F* ^2^)] = 0.041
*wR*(*F* ^2^) = 0.119
*S* = 1.07
2687 reflections
122 parameters
H atoms treated by a mixture of independent and constrained refinement
Δρ_max_ = 0.30 e Å^−3^
Δρ_min_ = −0.30 e Å^−3^

### 

Data collection: *COLLECT* (Nonius, 1998 ▶); cell refinement: *SCALEPACK* (Otwinowski & Minor, 1997 ▶); data reduction: *DENZO* (Otwinowski & Minor 1997 ▶) and *SCALEPACK*; program(s) used to solve structure: *SHELXS97* (Sheldrick, 2008 ▶); program(s) used to refine structure: *SHELXL97* (Sheldrick, 2008 ▶); molecular graphics: *SHELXTL* (Sheldrick, 2008 ▶); software used to prepare material for publication: *SHELXTL*.

## Supplementary Material

Crystal structure: contains datablocks I, global. DOI: 10.1107/S1600536810014200/ci5044sup1.cif

Structure factors: contains datablocks I. DOI: 10.1107/S1600536810014200/ci5044Isup2.hkl

Additional supplementary materials:  crystallographic information; 3D view; checkCIF report

## Figures and Tables

**Fig. 1.**
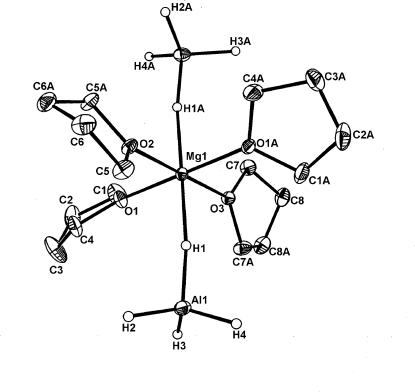
The molecular structure of [Mg(AlH4)2(C4H8O)4], showing 50% probability displacement ellipsoids and the atomic numbering scheme. Atoms labelled with the suffix A are generated by the symmetry operation (-x, 1/2-y, z).

## supplementary crystallographic information

### Comment

Mg(AlH_4_)_2_.4THF, (I), is a starting material for the synthesis of Mg(AlH_4_)_2_ which is an interesting candidate for hydrogen storage applications because of its high theoretical hydrogen storage capacity. Ashby *et al.* (1970) reported the synthesis of (I) by the metathesis reaction between NaAlH_4_ and MgCl_2_. Noth *et al.* (1995) and recently Fichtner & Fuhr (2002) reported the crystal structure of (I), but neither of the groups obtained high quality single crystal X-ray diffraction data. In the present work good quality single crystals were obtained from reaction between NaAlH_4_ and ClMgBH_4_ where the product, AlH_4_MgBH_4_.THF disproportionated to form (I). The crystal structure was determined using single crystal X-ray diffraction and compared with the previously reported data.

In general, the present crystal structure determination confirms the previous results. As previously described by Noth *et al.* (1995) and Fichtner & Fuhr (2002), the structure of (I) consists of discrete octahedral building blocks where four THF molecules and two tetrahedral AlH_4_^–^ units are connected to a Mg central atom. Fichtner & Fuhr (2002) reported only lattice parameters without coordinates of the atoms. Noth *et al.* (1995) reported the Al—H(t) and Al—H(b) bond lengths as 1.214 and 1.528 Å, respectively, which are shorter than expected. Moreover, the structure was only refined to a final R value of 0.065. We have redetermined this crystal structure at 150 K, with a final R value of 0.040 to obtain more precise data. In the present work, the Al—H(t) and Al—H(b) bond lengths were found to be 1.524 and 1.573 Å, respectively, which are close to the Al—H bond distance in other alanates. Al—H distances reported in other alanates with AlH_4_^–^ tetrahedral are 1.547 Å (at 8 K) for LiAlH_4_ (Sklar & Post, 1967), 1.532 Å (at 296 K) for NaAlH_4_ (Lauher *et al.*, 1979), 1.55 Å (at 200 K) for Mg(AlH_4_)_2_.Et_2_O (Fichtner & Fuhr, 2002) and 1.65 Å (at 230 K) for Ca(AlH_4_)_2_.4THF (Fichtner *et al.*, 2004).

### Experimental

All the manipulations were carried out in high vacuum lines and an Ar filled glove box to avoid the compounds reacting with oxygen and moisture. Solvents were dried by vacuum distillation from sodium benzophenone ketyl. Precursor ClMgBH_4_ was synthesized by ball milling MgCl_2_ and Mg(BH_4_)_2_ in 1:1 mole ratio in a high energy ball mill for 1 h. AlH_4_MgBH_4_ was prepared by the procedure reported by Ashby & Goel (1977). In a typical procedure, a clear solution of NaAlH_4_ in THF was added to a solution of ClMgBH_4_ in THF with rapid stirring for 60 min at room temperature. After completion of reaction, NaCl was filtered out from the solution and the solvent was removed from the filtrate under dynamic vacuum. The obtained AlH_4_MgBH_4_.THF powder was dissolved in benzene, filtered, concentrated, and aged for 2 days. AlH_4_MgBH_4_.THF slowly disproportionated to give colourless crystals of (I).

### Refinement

H atoms bonded to aluminium atoms were located and refined isotropically. The range of refined Al–H distances is 1.50 (2)–1.573 (18) Å. The remaining H atoms were placed in calculated positions [C–H = 0.99 Å] and refined using a rigid model with U_iso_(H) = 1.2U_eq_(C).

### Crystal data

**Table:**

[Al_2_MgH_8_(C_4_H_8_O)_4_]	*F*(000) = 824
*M**_r_* = 374.75	*D*_x_ = 1.063 Mg m^−^^3^
Orthorhombic, *P**c**n**b*	Mo *K*α radiation, λ = 0.71073 Å
Hall symbol: -P 2b 2ac	Cell parameters from 2687 reflections
*a* = 10.161 (2) Å	θ = 2.4–27.5°
*b* = 14.027 (3) Å	µ = 0.16 mm^−^^1^
*c* = 16.429 (3) Å	*T* = 150 K
*V* = 2341.6 (8) Å^3^	Cube, colourless
*Z* = 4	0.38 × 0.31 × 0.19 mm

### Data collection

**Table:**

Nonius Kappa CCD diffractometer	2687 independent reflections
Radiation source: fine-focus sealed tube	1973 reflections with *I* > 2σ(*I*)
graphite	*R*_int_ = 0.017
φ and ω scans	θ_max_ = 27.5°, θ_min_ = 2.4°
Absorption correction: multi-scan (*SCALEPACK*; Otwinowski & Minor, 1997)	*h* = −13→13
*T*_min_ = 0.940, *T*_max_ = 0.969	*k* = −18→18
5018 measured reflections	*l* = −21→21

### Refinement

**Table:**

Refinement on *F*^2^	Primary atom site location: structure-invariant direct methods
Least-squares matrix: full	Secondary atom site location: difference Fourier map
*R*[*F*^2^ > 2σ(*F*^2^)] = 0.041	Hydrogen site location: inferred from neighbouring sites
*wR*(*F*^2^) = 0.119	H atoms treated by a mixture of independent and constrained refinement
*S* = 1.07	*w* = 1/[σ^2^(*F*_o_^2^) + (0.0615*P*)^2^ + 0.6568*P*] where *P* = (*F*_o_^2^ + 2*F*_c_^2^)/3
2687 reflections	(Δ/σ)_max_ = 0.001
122 parameters	Δρ_max_ = 0.30 e Å^−^^3^
0 restraints	Δρ_min_ = −0.30 e Å^−^^3^

### Special details

**Table:**

Geometry. All esds (except the esd in the dihedral angle between two l.s. planes) are estimated using the full covariance matrix. The cell esds are taken into account individually in the estimation of esds in distances, angles and torsion angles; correlations between esds in cell parameters are only used when they are defined by crystal symmetry. An approximate (isotropic) treatment of cell esds is used for estimating esds involving l.s. planes.
Refinement. Refinement of *F*^2^ against ALL reflections. The weighted *R*-factor wR and goodness of fit *S* are based on *F*^2^, conventional *R*-factors *R* are based on *F*, with *F* set to zero for negative *F*^2^. The threshold expression of *F*^2^ > σ(*F*^2^) is used only for calculating *R*-factors(gt) etc. and is not relevant to the choice of reflections for refinement. *R*-factors based on *F*^2^ are statistically about twice as large as those based on *F*, and *R*- factors based on ALL data will be even larger.

### Fractional atomic coordinates and isotropic or equivalent isotropic displacement parameters (Å^2^)

**Table:**

	*x*	*y*	*z*	*U*_iso_*/*U*_eq_	
Al1	0.22387 (5)	0.44321 (4)	0.13620 (3)	0.03294 (17)	
Mg1	0.0000	0.2500	0.13728 (4)	0.02116 (19)	
O1	0.16625 (10)	0.16699 (8)	0.13767 (6)	0.0298 (3)	
O3	0.0000	0.2500	0.01068 (8)	0.0269 (3)	
O2	0.0000	0.2500	0.26391 (8)	0.0270 (3)	
C8	−0.06993 (19)	0.23112 (14)	−0.12525 (9)	0.0429 (5)	
H8A	−0.1341	0.2828	−0.1356	0.052*	
H8B	−0.0815	0.1806	−0.1667	0.052*	
C4	0.27768 (17)	0.17950 (14)	0.19205 (12)	0.0439 (5)	
H4A	0.2588	0.1515	0.2461	0.053*	
H4B	0.2982	0.2480	0.1991	0.053*	
C5	0.02463 (19)	0.33286 (12)	0.31515 (9)	0.0370 (4)	
H5A	−0.0285	0.3880	0.2967	0.044*	
H5B	0.1189	0.3507	0.3136	0.044*	
C7	−0.08466 (18)	0.19178 (13)	−0.04047 (9)	0.0382 (4)	
H7A	−0.1773	0.1962	−0.0222	0.046*	
H7B	−0.0570	0.1242	−0.0386	0.046*	
C6	−0.0151 (2)	0.30305 (13)	0.39968 (10)	0.0445 (5)	
H6A	−0.1098	0.3151	0.4094	0.053*	
H6B	0.0373	0.3370	0.4415	0.053*	
C2	0.3257 (2)	0.05289 (16)	0.10336 (13)	0.0573 (6)	
H2A	0.3781	0.0396	0.0538	0.069*	
H2B	0.3177	−0.0066	0.1354	0.069*	
C3	0.3878 (2)	0.1296 (2)	0.15261 (16)	0.0763 (8)	
H3A	0.4480	0.1022	0.1938	0.092*	
H3B	0.4382	0.1737	0.1174	0.092*	
C1	0.1945 (2)	0.09039 (16)	0.08170 (14)	0.0587 (6)	
H1A	0.1945	0.1143	0.0250	0.070*	
H1B	0.1272	0.0396	0.0865	0.070*	
H1	0.1142 (17)	0.3641 (12)	0.1382 (9)	0.034 (5)*	
H2	0.2892 (19)	0.4426 (13)	0.2215 (13)	0.055 (6)*	
H3	0.3167 (19)	0.4126 (16)	0.0687 (13)	0.063 (6)*	
H4	0.156 (2)	0.5361 (18)	0.1206 (14)	0.076 (7)*	

### Atomic displacement parameters (Å^2^)

**Table:**

	*U*^11^	*U*^22^	*U*^33^	*U*^12^	*U*^13^	*U*^23^
Al1	0.0350 (3)	0.0315 (3)	0.0323 (3)	−0.0085 (2)	−0.0024 (2)	0.0047 (2)
Mg1	0.0227 (4)	0.0223 (4)	0.0184 (3)	0.0009 (3)	0.000	0.000
O1	0.0284 (6)	0.0313 (6)	0.0297 (6)	0.0073 (5)	−0.0075 (4)	−0.0111 (4)
O3	0.0292 (8)	0.0336 (8)	0.0178 (7)	−0.0037 (6)	0.000	0.000
O2	0.0401 (9)	0.0208 (7)	0.0200 (7)	−0.0038 (7)	0.000	0.000
C8	0.0587 (12)	0.0459 (11)	0.0243 (8)	0.0041 (9)	−0.0085 (8)	−0.0006 (7)
C4	0.0391 (10)	0.0438 (10)	0.0488 (11)	0.0098 (8)	−0.0215 (8)	−0.0096 (9)
C5	0.0550 (11)	0.0294 (9)	0.0268 (8)	−0.0089 (8)	0.0003 (7)	−0.0075 (7)
C7	0.0442 (10)	0.0455 (10)	0.0249 (8)	−0.0094 (8)	−0.0090 (7)	−0.0014 (7)
C6	0.0556 (12)	0.0522 (12)	0.0257 (8)	−0.0139 (9)	0.0049 (8)	−0.0098 (8)
C2	0.0657 (14)	0.0601 (14)	0.0462 (11)	0.0366 (11)	−0.0033 (10)	−0.0097 (10)
C3	0.0344 (12)	0.107 (2)	0.0876 (17)	0.0248 (12)	−0.0139 (11)	−0.0386 (16)
C1	0.0526 (12)	0.0554 (13)	0.0681 (14)	0.0211 (10)	−0.0125 (10)	−0.0379 (11)

### Geometric parameters (Å, °)

**Table:**

Al1—H1	1.573 (18)	C4—C3	1.471 (3)
Al1—H2	1.55 (2)	C4—H4A	0.99
Al1—H3	1.52 (2)	C4—H4B	0.99
Al1—H4	1.50 (2)	C5—C6	1.505 (2)
Mg1—O1^i^	2.0517 (11)	C5—H5A	0.99
Mg1—O1	2.0518 (11)	C5—H5B	0.99
Mg1—O3	2.0800 (15)	C7—H7A	0.99
Mg1—O2	2.0804 (15)	C7—H7B	0.99
Mg1—H1	1.977 (18)	C6—C6^i^	1.519 (4)
O1—C1	1.443 (2)	C6—H6A	0.99
O1—C4	1.4529 (19)	C6—H6B	0.99
O3—C7^i^	1.4537 (17)	C2—C1	1.477 (3)
O3—C7	1.4537 (17)	C2—C3	1.487 (3)
O2—C5	1.4567 (17)	C2—H2A	0.99
O2—C5^i^	1.4567 (17)	C2—H2B	0.99
C8—C7	1.506 (2)	C3—H3A	0.99
C8—C8^i^	1.517 (4)	C3—H3B	0.99
C8—H8A	0.99	C1—H1A	0.99
C8—H8B	0.99	C1—H1B	0.99
			
H1—Al1—H2	106.3 (9)	O2—C5—C6	105.40 (13)
H1—Al1—H3	104.8 (10)	O2—C5—H5A	110.7
H2—Al1—H3	113.1 (11)	C6—C5—H5A	110.7
H1—Al1—H4	107.0 (11)	O2—C5—H5B	110.7
H2—Al1—H4	110.9 (11)	C6—C5—H5B	110.7
H3—Al1—H4	114.0 (12)	H5A—C5—H5B	108.8
O1^i^—Mg1—O1	179.65 (6)	O3—C7—C8	105.66 (13)
O1^i^—Mg1—O3	90.18 (3)	O3—C7—H7A	110.6
O1—Mg1—O3	90.18 (3)	C8—C7—H7A	110.6
O1^i^—Mg1—O2	89.82 (3)	O3—C7—H7B	110.6
O1—Mg1—O2	89.82 (3)	C8—C7—H7B	110.6
O3—Mg1—O2	180.0	H7A—C7—H7B	108.7
O1^i^—Mg1—H1	91.4 (5)	C5—C6—C6^i^	102.59 (11)
O1—Mg1—H1	88.6 (5)	C5—C6—H6A	111.2
O3—Mg1—H1	90.4 (4)	C6^i^—C6—H6A	111.2
O2—Mg1—H1	89.6 (4)	C5—C6—H6B	111.2
C1—O1—C4	109.08 (13)	C6^i^—C6—H6B	111.2
C1—O1—Mg1	125.75 (10)	H6A—C6—H6B	109.2
C4—O1—Mg1	125.10 (10)	C1—C2—C3	104.88 (16)
C7^i^—O3—C7	109.37 (16)	C1—C2—H2A	110.8
C7^i^—O3—Mg1	125.32 (8)	C3—C2—H2A	110.8
C7—O3—Mg1	125.32 (8)	C1—C2—H2B	110.8
C5—O2—C5^i^	109.39 (16)	C3—C2—H2B	110.8
C5—O2—Mg1	125.30 (8)	H2A—C2—H2B	108.8
C5^i^—O2—Mg1	125.30 (8)	C4—C3—C2	105.13 (18)
C7—C8—C8^i^	102.78 (11)	C4—C3—H3A	110.7
C7—C8—H8A	111.2	C2—C3—H3A	110.7
C8^i^—C8—H8A	111.2	C4—C3—H3B	110.7
C7—C8—H8B	111.2	C2—C3—H3B	110.7
C8^i^—C8—H8B	111.2	H3A—C3—H3B	108.8
H8A—C8—H8B	109.1	O1—C1—C2	106.93 (15)
O1—C4—C3	105.34 (15)	O1—C1—H1A	110.3
O1—C4—H4A	110.7	C2—C1—H1A	110.3
C3—C4—H4A	110.7	O1—C1—H1B	110.3
O1—C4—H4B	110.7	C2—C1—H1B	110.3
C3—C4—H4B	110.7	H1A—C1—H1B	108.6
H4A—C4—H4B	108.8		

Symmetry codes: (i) −*x*, −*y*+1/2, *z*.

## References

[bb1] Ashby, E. C. & Goel, A. B. (1977). *Inorg. Chem.* **16**, 2082–2085.

[bb2] Ashby, E. C., Schwartz, R. D. & James, B. D. (1970). *Inorg. Chem.* **9**, 325–332.

[bb3] Dilts, J. A. & Ashby, E. C. (1972). *Inorg. Chem.* **11**, 1230–1236.

[bb4] Fichtner, M., Frommen, C. & Fuhr, O. (2004). *Inorg. Chem* **44**, 3479–3484. 10.1021/ic048291q 15877429

[bb5] Fichtner, M. & Fuhr, O. (2002). *J. Alloys Compd*, **345**, 286–296.

[bb6] Lauher, J. W., Dougherty, D. & Herley, P. J. (1979). *Acta Cryst.* B**35**, 1454–1456.

[bb7] Nonius (1998). *COLLECT* Nonius BV, Delft, The Netherlands.

[bb8] Nöth, H., Schmidt, M. & Treitl, A. (1995). *Chem. Ber.* **128**, 999–1006.

[bb9] Otwinowski, Z. & Minor, W. (1997). *Methods in Enzymology*, Vol. 276, *Macromolecular Crystallography*, Part A, edited by C. W. Carter Jr & R. M. Sweet, pp. 307–326. New York: Academic Press.

[bb10] Sheldrick, G. M. (2008). *Acta Cryst.* A**64**, 112–122. 10.1107/S0108767307043930 18156677

[bb11] Shen, P. & Che, Y. (1991). *Faming ZhuanliShenqing Gongkai Shuomingshu*, CN1051179, p. 7.

[bb12] Sklar, N. & Post, B. (1967). *Inorg. Chem* **6**, 669–671.

